# Development and validation of anthropometric equations to estimate appendicular muscle mass in elderly women

**DOI:** 10.1186/1475-2891-12-92

**Published:** 2013-07-02

**Authors:** Piettra Moura Galvão Pereira, Giselma Alcântara da Silva, Gilberto Moreira Santos, Edio Luiz Petroski, Amandio Aristides Rihan Geraldes

**Affiliations:** 1 Laboratory of Physical Fitness, Performance and Health, Center for Physical Education and Sports Education Center, Federal University of Alagoas, Maceió-Alagoas, Brazil; 2 Graduate Program in Nutrition, Federal University of Alagoas, Maceió-Alagoas, Brazil; 3 Sports Center, Center for Research in Kinanthropometry and Human Performance, Federal University of Santa Catarina, Florianópolis, Santa Catarina, Brazil

**Keywords:** Aging, Sarcopenia, Skeletal muscle mass, Anthropometry, Body composition

## Abstract

**Objective:**

This study aimed to examine the cross validity of two anthropometric equations commonly used and propose simple anthropometric equations to estimate appendicular muscle mass (AMM) in elderly women.

**Methods:**

Among 234 physically active and functionally independent elderly women, 101 (60 to 89 years) were selected through simple drawing to compose the study sample. The paired t test and the Pearson correlation coefficient were used to perform cross-validation and concordance was verified by intraclass correction coefficient (ICC) and by the Bland and Altman technique. To propose predictive models, multiple linear regression analysis, anthropometric measures of body mass (BM), height, girth, skinfolds, body mass index (BMI) were used, and muscle perimeters were included in the analysis as independent variables. Dual-Energy X-ray Absorptiometry (AMM_DXA_) was used as criterion measurement. The sample power calculations were carried out by *Post Hoc Compute Achieved Power*. Sample power values from 0.88 to 0.91 were observed.

**Results:**

When compared, the two equations tested differed significantly from the AMM_DXA_ (p <0.001 and p = 0.001). Ten population / specific anthropometric equations were developed to estimate AMM, among them, three equations achieved all validation criteria used: AMM _(E2)_ = 4.150 +0.251 [bodymass (BM)] - 0.411 [bodymass index (BMI)] + 0.011 [Right forearm perimeter (PANTd) ^2^]; AMM _(E3)_ = 4.087 + 0.255 (BM) - 0.371 (BMI) + 0.011 (PANTd) ^2^ - 0.035 [thigh skinfold (DCCO)]; MMA _(E6)_ = 2.855 + 0.298 (BM) + 0.019 (Age) - 0,082 [hip circumference (PQUAD)] + 0.400 (PANTd) - 0.332 (BMI). The equations estimated the criterion method (p = 0.056 p = 0.158), and explained from 0.69% to 0.74% of variations observed in AMM_DXA_ with low standard errors of the estimate (1.36 to 1.55 kg) and high concordance (ICC between 0,90 and 0.91 and concordance limits from -2,93 to 2,33 kg).

**Conclusion:**

The equations tested were not valid for use in physically active and functionally independent elderly women. The simple anthropometric equations developed in this study showed good practical applicability and high validity to estimate AMM in elderly women.

## Introduction

The elderly population is increasing in most parts of the world, including in developing countries [1,2]. Data from the Brazilian Institute of Geography and Statistics [3] show that also in Brazil, there is an increase in the percentage of elderly individuals. According to [3], the northeastern region of Brazil showed an increase of 7.2% in the elderly population. It is estimated that within 20 years, the elderly population will correspond to approximately 13% of the Brazilian population. Such demographic outlook implies new international, national and local demands to improve the quality of life of this population [1].

With the aging process, important alterations can be observed, especially in body composition such as: height and body mass reduction and decreased muscle mass and as a consequence, reduced muscle strength. This condition is commonly called as sarcopenia [4,5].

Sarcopenia is considered an independent risk factor for frailty, functional impairment, falls and loss of independence in older subjects [6], and associated with worse health status and quality of life [7], social isolation [8], need for professional care and hospitalizations [9,10], increased morbidity and mortality rates from all causes [11,12], represent a public health problem with significant impacts on economy [10,13].

Evans and Rosenberg [14] highlight that no decline with age is so dramatic and potentially more significant than the decline in muscle mass. Although the highest losses of skeletal muscle mass are verified in men, it has been suggested that for women, sarcopenia is a major public health problem [15,16]. This assertion has been justified by the fact that women have lower muscle mass and strength when young and higher life expectancy, which implies the high rates of functional limitations in this gender [5,17]. Additionally, females exhibit greater vulnerability related to aging, due to physiologic exhaustion of the ovarian function (menopause) and hence reduced estrogen, thus enhancing the effects of sarcopenia [18,19].

Since appendicular muscle mass (AMM) is closely related to ambulation, mobility, and functional independence [15] and consequently with the performance of daily activities [8], the most important and significant muscle losses associated with aging are observed in the appendicular skeleton [7] and may show declines from 1 to 2% per year [20]. Thus, the maintenance of the AMM structure and function in the elderly is necessary to preserve mobility and functional independence [21].

Among the various techniques available to assess AMM in humans, MRI is the current gold standard [22,23], however, it has high cost, which limits its use in research and clinical practice [24]. An alternative technique is the Dual-energy X-ray Absorptiometry (DXA), which allows measuring the AMM validly when compared with other techniques such as Total Body Nitrogen [25] Nuclear Magnetic Resonance [26] and Computed Tomography [27]. In addition, for being a rapid and practical technique, DXA has been increasingly used in laboratory studies [26,28,29]. However, using DXA in field studies or on a large scale is impractical due to its high cost and logistics required to perform the measurements [30].

On the other hand, due to its characteristics, the anthropometric method has been a valid, accurate, innocuous and inexpensive alternative to measure body composition [31,32]. Recently, Kanellaris and Manios [32] performed the validation of simple anthropometric models to estimate body fat in postmenopausal women, which provides us with a two-compartment model for assessing body composition (fat mass and lean mass). However, for being a two-compartment model for assessing body composition, the predictive equations of body fat do not allow a specific measurement of the amount of muscle mass and especially the AMM for diagnosing sarcopenia. Therefore, several models of predictive equations to assess AMM have been developed in samples composed of individuals with specific characteristics [33-35]; therefore for use in a general way or in other populations, these models must be validated [33,36-38].

In this context, the present study aimed to verify the cross-validity of predictive equations proposed by two authors: Baumgartner et al. [33] and Tankó et al. [37] and propose simple anthropometric equations to estimate AMM in elderly women.

## Material and methods

### Population and sample

This cross-validation study was carried out using the database of a research project entitled: “*Assessing body composition in the elderly: a normative study*”. The study protocol was approved by the ethics committee on research with humans of the Federal University of Alagoas under number: 020.487/2008-53.

The number of subjects selected to compose the study sample was estimated considering the number of subjects used as subjects in cross-validation studies of anthropometric equations to assess lean and / or muscle mass in the elderly [31,39], and the sample size needed for this type of study was recommended by statistics textbooks [40,41].

The population of this study consisted of 234 physically active and functionally independent elderly women, among them, 101 were randomly selected to compose the sample and had all body composition assessment and interview data collected.

Subjects who reported diseases that could affect the musculoskeletal system (e.g., neuropathies, chronic obstructive pulmonary disease, active cancer or recent cancer treatment), those who used drugs that alter body composition such as corticosteroids, androgens or anti-androgen drugs and antipsychotics, those who have suffered myocardial infarction recently and those who had body mass exceeding 100 kg and / or bitrochanteric diameter wider than the DXA table (65 cm) were excluded from the study population. The information required for the evaluation of inclusion and exclusion criteria were obtained with the aid of the interview and / or anthropometric measurements.

The sample was distributed by three different groups: two validation groups (GV1 and GV2) and one estimate group (GE). Authors like Maroco [42] and Snee [43] suggested that when attempting to validate a model, one should always use a set of data different from that used for its development, where 60% of the sample data should be used in model adjustment and the other 40% in its validation.

GV1 was used to perform the cross-validation attempt of two equations: Baumgartner et al. [33] and Tankó et al. [37] composed of 84 subjects who had performed the handgrip strength test, since this is an independent variable in the equation of Baumgartner et al. [33] to perform cross-validation. GE (n = 60) was used to develop new anthropometric equations to estimate AMM and GV2 (n = 41) was used to validate the anthropometric equations developed; the participation of subjects in these two latter groups were mutually exclusive.

### Anthropometric measures

The protocol recommended by Lohman et al. [44] was used to assess all anthropometric measures.

The independent variables collected were: body mass (BM), measured in kilograms (kg) with the aid of a digital scale (Plena® MEA-07400, *Measurement Specialites*, Inc, USA) with sensitivity of 100 g; stature (ST) measured in meters (m) with the aid of a portable stadiometer (Seca®, Baystate Scale & Systems, USA) with sensitivity of 0.1 millimeters (mm); body mass index (BMI) was calculated by dividing body weight by the squared height (kg / m^2^); triceps, biceps, subscapularis, midaxillary, suprailiac, abdomen, thigh (TS) and leg skinfold thickness measurements were measured in millimeters (mm) and assessed at the right hemisphere with the aid of calipers label Lange (Beta Technology Incorporated, Cambridge, Maryland, USA) with accuracy of 0.1 mm; right forearm (PANTd), left forearm, right arm, left arm, waist, abdomen, hip (PH), right thigh, left thigh, right leg and left leg body perimeter were measured in centimeters (cm) with the aid of a metal, flexible and inelastic tape measure label Sanny (American Medical do Brasil Ltda., São Bernardo do Campo, SP), with accuracy of 0.1 mm.

Anthropometric measurements were performed by four trained evaluators. The inter-rater and intra-rater reproducibility, respectively, for anthropometric variables held in a group of 17 subjects showed intraclass correlation coefficients from 0.83 to 0.98 for skinfold thickness measurements and from 0.76 to 0.98 for body perimeter measures.

Additionally, for muscle circumference in centimeters (cm) the following equation was used: Circ_muscular_ = Circ_limb_- (π x Skinfold) used in similar studies [35,45,46]. To calculate waist and abdomen muscle circumference, body perimeter waist and body perimeter of abdomen and skinfold thickness of suprailiac and skinfold thickness of abdomen measures were used, respectively. Since body perimeters were collected in both hemibodies to calculate appendicular skeletal muscle circumferences, arms: the mean body perimeter of right arm and left and skinfold thickness of triceps and skinfold thickness of biceps values were calculated; thighs (TMC): the mean body perimeter of right thigh and left and skinfold thickness of thigh values were used; legs: the mean body perimeter of leg right and left values were calculated and the skinfold thickness of leg was used.

### Body composition assessment

The body composition measurement was performed using a scanner label Lunar (Model: Prodigy Advance - DPX-YZB/2099 series; Madison, WI. Software 3.0) in a specialized clinic. All measurements were performed by the same technician and as recommended by the manufacturer, the device was calibrated daily as described in the manual.

The AMM was determined by the sum of soft lean tissue of upper and lower limbs, as proposed by Heymsfield et al. [25] and Baumgartner et al. [47]. Additionally, for descriptive purposes; body fat percentage (%BF) was determined by the total fat tissue; total mass muscle was estimated by equation proposed by Kim et al. [24] and appendicular muscle mass index was calculated similarly to BMI by dividing the appendicular muscle mass by the squared height (kg / m^2^), as recommended by Baumgartner et al. [33].

### Handgrip strength measurement

For being one of the variables used in the prediction equation proposed by Baumgartner et al. [33], the handgrip strength was measured with the aid of a manual hydraulic dynamometer label JAMAR (Hydraulic Hand Dynamometer® Model PC-5030 J1, Fred Sammons, Inc., Burr Ridge, IL: USA), following protocol recommended by the American Association of Hand Therapists [48].

### Anthropometric equations

Both equations used to verify the cross-validity: Baumgartner et al. [33] and Tanko et al. [37] could be observed in the table below (Table 1).

**Table 1:** Equations tested with the validation results and methods used by the authors

**Equations**	**Method**	**Model**	**R**^**2**^	**SEE**
Baumgartner *et al..* (1998) [33]	DXA	AMM = 0.2487(BM) + 0.0483(ST)-0.1584(GQUAD) +0.0732(HGS) +2.5843(SEX) + 5.8828	0.91	1.58
Tankó *et al.* (2002) [37]	DXA	AMM = −13.3-0.05(AGE) + 0.11(BM) + 16.1*(ST)	0.58	1.70

Abbreviation: *R*^*2*^ Determination coefficient, *SEE* (kg) Standard error of estimate, *DXA,* Dual Energy Absorptiometry Radiology, *AMM,* Appendicular skeletal muscle mass, *BM,* Body mass , *ST,* Height, *HP,* Hip circumference, *HGS,* Handgrip strength. Sex (0 = women).

### Statistical treatment

Data normality was verified using the Kolgomorov - Smirnov test corrected by Lilliefors and the residue variance homogeneity was verified using the Levene test. Mean, standard deviation and range were used to describe the anthropometric characteristics of the sample.

Depending on the type of distribution assigned to data, the Pearson and / or Spearman correlation coefficients were used to assess the association between anthropometric variables and AMM.

To achieve the objective of this study, statistical analysis was divided into two stages: Stage 1 and 2.

*Stage 01* - To perform the cross-validation, AMM results were verified by equations tested in GV1 and individually compared with DXA criterion measurement (AMM_DXA_) using the paired t test. Additionally, regression (R) and determination coefficients (R^2^) were verified and estimate standard, constant and total errors were calculated.

To consider equations as valid, the validation criteria recommended by Lohman [49] were used. That is, the results obtained by the equations tested and the criterion method should not present significant differences, standard error of estimate should be less than 3.5 and, finally, R^2^ should be greater than 0.7.

*Stage 02* - Given the existence of significant differences between methods for the development of regression equations to estimate AMM, multiple linear regression analyses were carried out in the GE with selection of variables through Stepwise and Enter methods. The latter method verified the assumptions to apply the regression models to adjust practical variables for use in the models developed.

The collinearity between variables was verified by the variance inflation factors (VIF) and tolerance (T) values. Thus, VIF values lower than 5 or even 10 were considered acceptable, as well as tolerance values above 0.1 [42,43].

The GE and GV2 results were compared by the independent Student’s t test with variables normalized, by the Mann Whitney test for variables that did not meet the normality criteria. Finally, after the end of the second stage, all steps in the first stage were performed this time with GV2 in each of the equations developed in this study.

To verify the concordance, Bland and Altman plots were performed [50] and the Intraclass correlation coefficient (ICC) was calculated with equations that met the cross-validation criteria adopted.

After checking the validation criteria adopted, the calculations of the sample power were performed by the *Post Hoc Compute Achieved Power* analysis using the G * Power software version 3.0.10. [51]. The other statistical calculations were performed with the aid of statistical package Statistical Package for Social Science, version SPSS® 12.0 (Chicago, IL, USA). A significance level of p <0.05 was adopted.

## Results

The descriptive characteristics of variables observed in the sample, as well as the correlations between independent variables and AMM_DXA_ and comparison between means of the independent variables for GE and GV2 are shown in Table 2.

**Table 2:** Descriptive characteristics, correlations observed between dependent variable with independent variables and comparison between groups

**Variables**	**Validation group 01**	**Estimation group**	**Validation group 02**	**AMM*x*Variables**		***p***
	**(n = 84)**	**(n = 60)**	**(n = 41)**	**(n = 101)**		
				**R**	**Rho**	
Age (years)	67.30 ± 6.24	66.75 ± 5.81	68.49 ± 6.59	−0.18	-	0.164
Body mass (kg)	64.05 ± 10.20	64.34 ± 10.06	62.55 ± 10.40	0.76	-	0.390
Height (m)	1.50 ± 0.06	1.50 ± 0.05	1.51 ± 0.06	0.61	-	0.554
BMI (kg m^-2^)	28.20 ± 4.07	28.50 ± 4.20	27.37 ± 3.88	0.47	-	0.174
AMM_BAUM_ (kg)	7.29 ± 1.80	-	-	-	-	-
AMM_TANK_ (kg)	14.64 ± 1.90	-	-	-	-	-
BF_DXA_ (%)	41.58 ± 6.51	42.20 ± 6.51	39.93 ± 6.27*	0.16	-	0.084
TMM _KIM_ (kg)	16.70 ± 2.81	16.56 ± 2.67	16.93 ± 2.98	0.99	-	0.524
AMM_DXA_ (kg)	15.15 ± 2.46	14.98 ± 2.34	15.33 ± 2.61	-	-	0.481
AMMI _DXA_ (kg m^-2^)	6.65 ± 0.82	6.62 ± 0.83	6.69 ± 0.85	0.86	-	0.651
Right arm ^P^ (cm)	29.70 ± 3.53	29.75 ± 3.67	29.35 ± 6.27	0.54	-	0.585
Left arm ^P^ (cm)	29.50 ± 3.63	29.64 ± 3.83	28.98 ± 3.48	0.54	-	0.382
Right forearm ^P^ (cm)	24.12 ± 1.67	24.10 ± 1.73	23.88 ± 1.97	0.70	-	0.553
Left forearm ^P^ (cm)	24.01 ± 2.09	23.98 ± 2.24	23.72 ± 2.08	0.68	-	0.547
Right leg ^P^ (cm)	35.26 ± 3.52	35.26 ± 3.83	35.07 ± 2.70	-	0.56	0.782
Left leg ^P^ (cm)	35.10 ± 3.41	35.16 ± 3.81	34.93 ± 2.51	-	0.53	0.716
Right thigh ^P^ (cm)	49.47 ± 8.84	49.75 ± 9.74	48.75 ± 5.72	-	0.48	0.555
Left thigh ^P^ (cm)	49.58 ± 7.58	49.51 ± 8.15	50.23 ± 6.78	0.53	-	0.647
Abdomen ^P^ (cm)	95.53 ± 12.52	96.34 ± 13.74	94.09 ± 7.68	-	0.34	0.299
Waist ^P^ (cm)	85.33 ± 9.15	85.91 ± 8.99	84.16 ± 8.08	0.45	-	0.320
Hip ^P^ (cm)	102.50 ± 8.52	102.66 ± 8.66	100.37 ± 7.46	0.42	-	0.170
Triceps ^S^ (mm)	27.67 ± 7.95	27.22 ± 8.57	24.76 ± 5.00	0.20	-	0.094
Biceps ^S^ (mm)	19.16 ± 7.98	20.08 ± 8.53	16.75 ± 5.92	0.25	-	0.053
Subscapularis ^S^ (mm)	28.04 ± 9.40	28.77 ± 9.34	26.37 ± 8.81	0.25	-	0.198
Axillary ^S^ (mm)	26.13 ± 8.46	26.06 ± 8.15	25.27 ± 8.38	0.19	-	0.796
Suprailiac ^S^ (mm)	32.13 ± 9.40	33.03 ± 9.64	30.00 ± 7.95	0.17	-	0.100
Abdomen ^S^ (mm)	38.27 ± 9.65	38.85 ± 10.38	36.35 ± 8.60	0.20	-	0.206
Thigh ^S^ (mm)	37.77 ± 12.30	37.65 ± 13.35	37.49 ± 10.68	0.07	-	0.950
Leg ^S^ (mm)	24.45 ± 8.60	24.97 ± 9.14	23.49 ± 6.90	0.02	-	0.384
Arm ^M^ (cm)	22.39 ± 2.56	22.27 ± 2.74	22.65 ± 2.24	-	0.54	0.467
Thigh ^M^ (cm)	37.67 ± 7.43	37.81 ± 8.02	37.71 ± 5.28	-	0.57	0.946
Leg ^M^ (cm)	27.50 ± 3.59	27.37 ± 3.89	27.62 ± 2.43	0.55	-	0.690
Waist ^M^ (cm)	75.24 ± 8.09	75.53 ± 8.34	74.73 ± 6.56	0.45	-	0.593
Abdomen ^M^ (cm)	83.51 ± 11.72	84.14 ± 12.92	82.68 ± 7.14	-	0.33	0.471
Handgrip (kg)	21.35 ± 5.21	21.03 ± 4.99	-	0.50	-	-

Abbreviation: *BMI,* Body mass index, *AMM*_*BAUM*_, Appendicular muscle mass equation estimated by equation of Baumgartner *et al*. *AMM*_*TANK*_, Appendicular muscle mass estimated by equation of Tankó *et al*. *BF*_*DXA*_, Body fat percentage estimated by absorptiometry, *TMM*_*KIM*_, Total muscle mass estimated by equation of Kim et al. *AMM*_*DXA,*_ Appendicular muscle mass estimated by DXA, *AMMI*_*DXA*_, Appendicular muscle mass index ^*P*^ Body perimeter, ^*S*^ Skinfold thickness, ^*M*^ Muscle circumference, *R,* Pearson correlation coefficient, *Rho*, Spearman correlation coefficient, *p,* probability observed in the comparison between groups.

* Significant difference p <0.05.

When comparing the mean of independent variables of GE and GV2, these did not differ from each other, indicating that the samples are statistically similar to perform the cross-validation.

Table 3 shows the results obtained in the cross-validation process for equations of Baumgartner et al. [33] and Tankó et al. [37]. When individually compared with DXA by the paired t test, AMM estimated by both equations tested, despite showing high correlations, significantly differed from values obtained by the criterion method: DXA.

**Table 3:** **Cross validation between equations of Baumgartner*et al*., Tanko*et al*. and criterion method**

**Equations**	**M ± SD**	***t***	***P***	**R**	**R**^**2**^	**EC (kg)**	**SEE (kg)**	**TE (kg)**
Baumgartner *et al.,* (1998) [33]	7.29 ± 1.80*	53.988	<0.001	0.84	0.71	−7.87	1.32	7.98
Tankó *et al.* (2002) [37]	14.64 ± 1.90*	3.297	0.001	0.80	0.65	−0.52	1.46	1.53
**AMM**_**DXA**_	15.16 ± 2.43	-	-	-		-	-	-

Abbreviation: M ± SD, Mean and standard deviation, *t* Paired t test, *p,* Probability observed in the comparison between groups, *R,* Regression coefficient, *R*^*2*^ Determination coefficient, *EC*, Error constant, *SEE,* Standard error of estimate, *TE,* Total error, *AMM*_*DXA*_, Appendicular muscle mass obtained by dual energy x-ray absorptiometry.

* Significant at p <0.05.

The models developed to estimate AMM_DXA_ can be seen in Table 4. It could be observed that none of the equations developed showed significant collinearity between independent variables.

**Table 4:** Models of equations developed to estimate appendicular muscle mass in elderly

**Model**	**Equation**	**R**	**R**^**2**^	**R**^**2**^_**a**_	**SEE**	***T***	**VIF**
**1**^**S**^	AMM = 5.843 + 0.309(BM)-0.376(BMI)					0,277	3,611
		0.83	0.70	0.68	1.33		
						0,277	3,611
**2**^**S**^	AMM = 4.150 + 0.251(BM)-0.411(BMI) + 0.011(PANTd)^2^					0,215	4,643
		0.87	0.75	0.73	1.21	0,272	3,678
						0,393	2,544
**3**^**S**^	AMM = 4.087 + 0.255(BM)-0.371(BMI) + 0.011(PANTd)^2^-0.035(TS)					0,215	4,649
						0,261	3,833
		0.88	0.78	0.77	1.14		
						0,393	2,544
						0,818	1,223
**4**^**S**^	AMM = 7.944 + 0.244(BM) + 0.010(AGE)-0.145(PH) + 0.230(PANTd)					0,189	5,249
						0,905	1,105
		0.84	0.70	0.68	1.34		
						0,389	2,573
						0,332	3,009
**5**^**E**^	AMM = 5.927 + 0.2399(BM) + 0.0119(AGE)-0.121(PH) + 0.272(PANTd)-0.033(TS)					0,188	5,318
						0,905	1,105
						0,302	3,307
		0.85	0.73	0.70	1.29		
						0,383	2,608
						0,768	1,301
**6**^**E**^	AMM = 2.855 + 0.298(BM) + 0.019(AGE)-0.082(PH) + 0.400(PANTd)-0.332(BMI)					0,167	6,004
						0,212	4,719
		0.88	0.77	0.75	1.17	0,901	1,110
						0,359	2,785
						0,268	3,725
**7**^**E**^	AMM = 11.631 + 0.256(BM)-0.141(PH) + 0.036(TMC)					0,295	3,388
		0.84	0.70	0.68	1.32	0,356	2,806
						0,736	1,358
**8**^**E**^	AMM = 3.971 + 0.292(BM)-0.328(BMI) + 0.397(PANTd)-0.078(PH)					0,176	5,690
						0,213	4,694
		0.88	0.77	0.75	1.17		
						0,359	2,782
						0,280	3,574
**9**^**E**^	AMM = 8.527 + 0.230(PANTd)-0.142(PH) + 0.241(BM)	0.84	0.70	0.68	1.33	0,198	5,048
						0,389	2,573
						0,356	2,810
**10**^**E**^	AMM = 6.575 + 0.272(PANTd)-0.117(PH) + 0.236(BM)-0.033(TS)					0,197	5,069
						0,383	2,608
		0.85	0.72	0.70	1.28		
						0,321	3,115
						0,769	1,301

Abbreviation: *R,* Correlation coefficients, *R*^*2*^ Determination coefficient, *R*^*2*^_*a*_ Adjusted determination coefficient, *SEE,* Standard error of estimate, *T,* Tolerance, *VIF,* Variance inflation factors, *BM* Body mass, *BMI,* Body mass index, *PANTd* Right forearm perimeter, *PANTd*^*2*^, Squared right forearm perimeter, *PH,* Hip perimeter, *TS,* Thigh skinfold, *TMC,* Thigh muscle circumference, ^*S*^ Stepwise, ^*E*^ Enter.

The results obtained in the cross-validation process of equations developed and DXA are described in Table 5. The equations of number two (E2), three (E3) and six (E6) met all validation criteria generally used in similar studies [31,47].

**Table 5:** Cross-validation of anthropometric equations developed

**Model**	**M ± SD**	***t***	***P***	**R**	**R**^**2**^	**R**^**2**^_**a**_	**ICC**	**EC**	**SEE**	**TE**
**1**	14.88 ± 2.10*	2.049	0.047	0.84	0.71	0.70	0,89	−0.45	1.52	1.47
**2**	14.92 ± 2.42	1.844	0.073	0.83	0.69	0.68	0,90	−0.46	1.55	1.49
**3**	14.86 ± 2.32	1.970	0.056	0.83	0.69	0.69	0,90	−0.45	1.47	1.51
**4**	14.83 ± 2.04*	2.133	0.039	0.82	0.67	0.66	0,88	−0.50	1.52	1.57
**5**	14.74 ± 2.15*	2.455	0.019	0.81	0.65	0.65	0,88	−0.59	1.55	1.63
**6**	15.03 ± 2.20	1.440	0.158	0.86	0.74	0.73	0,91	−0.30	1.36	1.36
**7**	14.84 ± 1.97	1.981	0.055	0.80	0.63	0.62	0,87	−0.49	1.60	1.64
**8**	14.91 ± 2.20	2.017	0.050	0.86	0.74	0.73	0,90	−0.60	1.35	1.39
**9**	14.84 ± 2.05*	2.084	0.044	0.82	0.67	0.66	0,88	−0.49	1.52	1.57
**10**	14.85 ± 2.15	2.003	0.052	0.81	0.65	0.65	0,88	−0.48	1.55	1.59
**AMM**_**DXA**_	15.33 ± 2.61	-	-	-	-	-	-	-	-	-

Abbreviation: M ± SD, Mean and standard deviation, *t* Paired t test, *p* Observed probability of the t test, *R,* Correlation coefficient, *R*^*2*^ Determination coefficient, *R*^*2*^_*a*_ Adjusted determination coefficient, *ICC,* Intraclass correlation coefficient, *EC,* Error constant, *SEE,* Standard error of estimate, *TE,* Total error, *AMM*_*DXA*_, Appendicular skeletal muscle mass measured by dual energy x-ray absorptiometry, * Significant difference (p <0.05).

The *post hoc* test to calculate the sample power of valid equations was conducted by adopting an error probability of 5% for the sample size used. The sample power (1-β err prob) was 0.91 (for E2 with three explanatory variables), 0.88 (for E3 with four explanatory variables) and 0.85 (for E6 with five explanatory variables).

**Figure 1:**
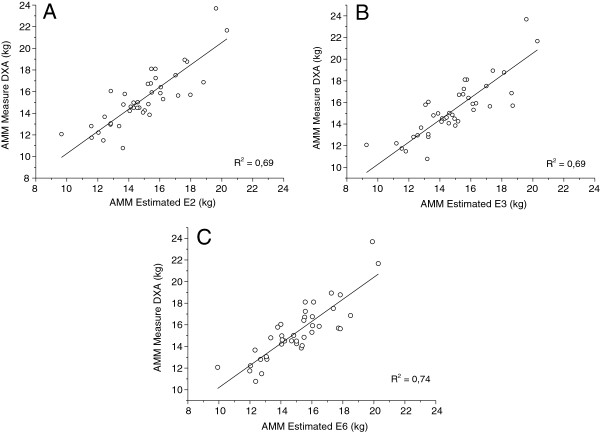
**Linear regression between equations developed and criterion method.** E2 = equation 02; E3 = equation 03; E6 = equation 06; AMM_DXA_ = dual energy X-ray absorptiometry.

The linear regression plotting between the dependent variable and the equations developed to estimate AMM validated in this study (Figure 1) indicates high predictive capacity of equations E2, E3 and E6 with correlation coefficients ranging from 0.69 to 0.74.

**Figure 2:**
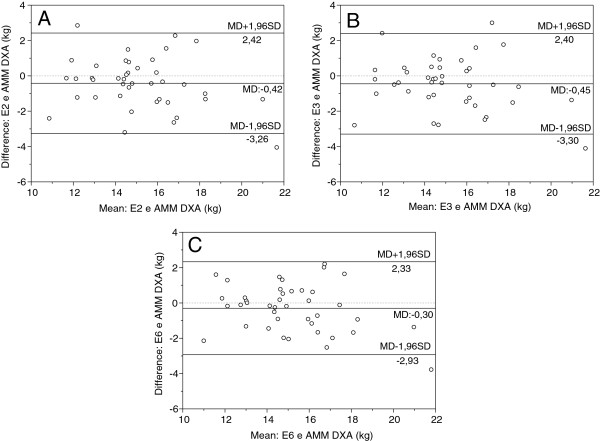
**Agreement between equations developed and criterion method**. E2 = equation 02; E3 = equation 03; E6 = equation 06; AMM_DXA_ = dual energy X-ray absorptiometry; MD = Mean differences, SD = standard deviation.

The agreement between AMM estimated by equations and DXA were tested using the Bland and Altman plotting, are shown in Figure 2. Equations 02, 03 and 06 showed mean errors from −0.30 to −0.45 kg and agreement limits from −2.93 to 2.33 kg.

## Discussion

The present study was carried out to verify the cross validity of two of the most widely used anthropometric equations for estimating AMM in the elderly, the equations of Baumgartner et al. [33] and Tankó et al. [37]. As it was not possible to validate the equations observed, new and simple regression equation models were developed and validated using anthropometric measurements to estimate AMM in a sample of apparently healthy and functionally independent elderly women using AMM_DXA_ as criterion measure.

Although the equations of Baumgartner et al. [33] and Tankó et al. [37] presented high correlation with AMM_DXA_, respectively: R = 0.84 and R = 0.80, they significantly differed from the criterion method (p <0.001 and p = 0.001, respectively). Therefore, in this study, 10 possible anthropometric equations for estimating AMM_DXA_ were developed. Among them, three stood out for not showing any significant difference with the criterion method (p between 0.056 and 0.158) due to the high correlation (R between 0.83 and 0.86) and concordance (ICC between 0.90 and 0.91 and concordance limits from −2.93 to 2.33 kg) with AMM_DXA_.

Baumgartner et al. [33] found sarcopenia prevalence in New Mexico. To this end, the authors developed an anthropometric equation to estimate AMM using AMM_DXA_ as criterion measure in a sub-sample of 199 physically active elderly subjects of both genders. The subjects were divided into two groups: estimation group (GE = 149 subjects) and validation group (GV = 50 subjects).

In that study, AMM predicted by the proposed equation, did not differ statistically from values measured by DXA, showing high correlation (R^2^ = 0.86) and small standard error of estimate (1.72 kg) between techniques. However, in this study, AMM verified by equation of Baumgartner et al. [33] (AMM_BAUM_), despite showing high correlation (R^2^ = 0.71) and adequate standard error of estimate (1.32 kg), differed statistically from criterion measure results: AMM_DXA_ (p <0.001). Moreover, the constant error of −7.87 kg indicated a strong tendency toward underestimation of AMM_DXA_ values and a quite high total error (7.98 kg), thus invalidating, in samples with characteristics similar to the present study (Table 03), the use of the proposed equation.

Tankó et al. [37], using a sample composed of 754 Danish women (17 to 85 years), verified which variables would best explain the variations of AMM and the upper limb muscle mass, estimated by DXA.

Among several independent variables considered in that study, age, BM and ST significantly contributed to variations of muscle parameter, being responsible for explaining 58% of the variance in AMM_DXA_ (R^2^ = 0.58), with moderate correlation coefficient (R = 0.76) and standard error of estimate of 1.70 kg. When the cross-validation of this study was performed (Table 03), AMM verified by the equation of Tankó et al. [37] (AMM_TANK_) showed correlation (R = 0.80) and determination coefficients (R^2^ = 0.65) higher than those observed in the original study sample, with low standard error of estimate, constant error and total error: 1.46 kg, -0.52 kg and 1.53 kg respectively. However, when compared, AMM_DXA_ and AMM_TANK_ showed statistically significant differences (p = 0.001). Therefore, the second equation did not meet the validation criteria adopted.

The statistical differences found between methods may be related to morphological differences observed in samples from the three studies: this study, the study of Baumgartner et al. [33] and that of Tankó et al. [37]. Baumgartner et al. [33], for example, did not characterize the samples of both groups: the development and validation of the equation, describing only the mean values of the overall sample composed of 833 individuals. Moreover, Tankó et al. [37] presented the physical characteristics of the subjects, divided into six age groups, where mean and standard deviation of age ranged from 25.7 ± 2.5 to 75.2 ± 3.4, BM from 62.9 ± 7.7 to 67.6 ± 10.01, ST from 1.59 ± 0.06 to 1.68 ± 0.06 and AMM from 19.4 ± 2.3 to 15.7 ± 2.4; however, such a comparison can be problematic, since the equation used to estimate AMM was developed using the total study sample, where, among the 754 participants, only 152 subjects were older than 60 years.

Due to considerable differences between the populations assessed, it is difficult to make valid comparisons between results found in the three studies. However, it appears that the AMM values measured in the present study (15.16 ± 2.43) were close to those observed by Baumgartner et al. [33] in their total sample (14.2 ± 1.9) and among the age groups 60–69 and > 70 years of subjects from the study of Tankó et al. [37] (16.5 ± 2.13 and 15.7 ± 2.4).

In recent research conducted in the city of São Paulo (southeastern Brazil), Gobbo et al. [52] found and described normative values for total muscle mass AMM and total and appendicular muscle mass indexes stratified by sex and age groups. To achieve their goals, the authors used the equation of Baumgartner et al. [33] to estimate AMM. However, the use of this equation was not preceded by cross-validation analysis, which very likely may raise doubts about the possible inadequacies of inferences performed in that study. In fact, so far, other Brazilian studies that have verified the validity of anthropometric equations proposed by Baumgartner et al. [33] and Tankó et al. [37] were not found.

In the present study, with the aid of multiple linear regression analyses, 10 models of anthropometric equations were developed (Table 03). Among these equations, six did not differ from the criterion method (Table 04).

Equations E2, E3 and E6 explained from 69% to 74% variations in AMM_DXA_ (Figure 1), reaching all validation criteria used. These models showed high correlation coefficients with the criterion method, ranging from 0.83 to 0.86, similar to the study of Baumgartner et al. [33], and higher than correlation found by Tanko et al. [37]. Moreover, the prediction errors observed in this study were lower than those observed in New Mexico and Denmark.

As for the analysis of concordance, both the ICC as the Bland and Altman analysis showed satisfactory values, indicating the possibility of using the equations developed and validated in this study. The ICC showed high values (E2 = 0.90, E3 = 0.90 and E6 = 0.91) showing a strong concordance with the DXA criterion method. The limits of the confidence intervals observed in valid models: E2 (2.42, -3.26 kg) E3 (−3.30, 2.40 kg) and E6 (−2.93, 2.33 kg) illustrated in Figure 2, were lower than those observed by Baumgartner et al. [33] (−5.1, 4.2 kg). Tankó et al. [37] in turn, did not use any statistical tool to verify the agreement.

The sample power (1-β errprob) calculated by the *post hoc* test in the three equations also appeared to be appropriate by adopting a confidence level of 95% for the sample size used. Thus, the probability of not making a type II error was 0.91, 0.88, and 0.85 for E2, E3 and E6, respectively.

Despite the limitations of this study, for example, the fact that the elderly women that composed the sampled showed homogeneity in relation to anthropometric characteristics, habits and physical skills, the three equations that showed the best conditions for use were therefore selected: E2, E3 and E6, because besides showing high validity, used variables of easy access. Characteristics necessary for the development of strategies to maintain or improve health, independence and quality of life in subjects with sarcopenia.

However, each model has its own advantages. For example, E2 has simple measures such as BM, BMI and the appendicular skeleton perimeter (PANTd) as independent variables, characteristic necessary in some situations of research and / or evaluation of body composition in non-laboratory conditions with the purpose of enabling a lower exposure of body parts and minimizing measurement errors due to the use of inadequate clothing; E3 uses BM, BMI, PANTd and one skinfold thickness measure (TS), E6 has the advantage of considering age as independent variable, This can be useful when evaluating AMM in a sample of elderly individuals with larger age ranges.

Moreover, the explanatory variables of AMM_DXA_ (BM, BMI, age, DCCO, PANTd, PQUAD) are easily mensured. Thus, as Baumgartner et al. [33], PQUAD was an explanatory variable of AMM_DXA_, and we can assume that this fact is related to the volume of muscles that make up the hip joint and responsible for the movements of the lower limbs (flexors, extensors, adductors, abductors and medial and lateral rotators of the hip).

The use of valid equations in combination with simple anthropometric models to assess BF% in older women is suggested as a strategy to identify subjects with sarcopenia, obesity and sarcopenic obesity, caused by the accumulation of intramuscular fat.

## Conclusion

Both equations used to estimate appendicular mass in older women verified in this study did not adequately meet the cross-validation criteria used as a reference. Therefore, they were not valid for use in elderly populations with the same characteristics as those who participated in this study. Moreover, among the equation models proposed in the study, using data from the estimation group and submitted to cross-validation in subjects from the validation group, models E2, E3 and E6, besides using simple access measures (bodyweight, age, body mass index, body circumferences and skinfold thickness), have satisfactory predictive capacity and are therefore suitable for use as a practical method for quantifying appendicular muscle mass in elderly women.

## Abbreviations

%BF: Body fat percentage; AMMBAUM: Appendicular muscle mass estimated by the equation of Baumgartner et al.; AMMDXA: Appendicular muscle mass estimated by dual energy X-ray absorptiometry; AMMI: Appendicular muscle mass index; AMM TANK: Appendicular muscle mass estimated by the equation of Tanko et al.; BM: Body mass; BMI: Body mass index; DXA: Dual-energy X-ray absorptiometry; EG: Estimate group; VG1: Validation group 01; VG2: Validation group 02; HGS: Hand grip strength; ICC: Intraclass correction coefficient; AMM: Appendicular muscle mass; PANTd: Right forearm perimeter; PH: Hip perimeter; NMR: Nuclear Magnetic Resonance; ST: Height; T: Tolerance; TMC: Thigh muscle circumference; TS: Skinfold thigh; VIF: Variance inflation factor.

## Competing interest

There are no conflicts of interest among authors.

## Authors’ contributions

PMGP contributed to statistical analysis, data interpretation, and manuscript writing. GAS contributed to statistical analysis, data interpretation, and manuscript writing. GMSJ contributed to study design and data collection. ELP contributed to data interpretation, critically reviewing the manuscript for important intellectual content. AARG Contributed to statistical analysis, data interpretation, manuscript writing, critically reviewing the manuscript for important intellectual content. All authors have read and approved the final manuscript.
